# Silicon µPCR Chip for Forensic STR Profiling with Hybeacon Probe Melting Curves

**DOI:** 10.1038/s41598-019-43946-5

**Published:** 2019-05-14

**Authors:** Senne Cornelis, Olivier Tytgat, Maarten Fauvart, Yannick Gansemans, Ann-Sophie Vander Plaetsen, Rodrigo S. Wiederkehr, Dieter Deforce, Filip Van Nieuwerburgh, Tim Stakenborg

**Affiliations:** 1 Laboratory of Pharmaceutical Biotechnology, Ghent University, 9000 Gent, Belgium; 2 Department of Life Science Technologies, Imec, 3001 Leuven, Belgium

**Keywords:** Genomics, Genomics

## Abstract

The demand to perform forensic DNA profiling outside of centralized laboratories and on the crime scene is increasing. Several criminal investigations would benefit tremendously from having DNA based information available in the first hours rather than days or weeks. However, due to the complexity and time-consuming nature of standard DNA fingerprinting methods, rapid and automated analyses are hard to achieve. We here demonstrate the implementation of an alternative DNA fingerprinting method in a single microchip. By combining PCR amplification and HyBeacon melting assays in a silicon Lab-on-a-chip (LoC), a significant step towards rapid on-site DNA fingerprinting is taken. The small form factor of a LoC reduces reagent consumption and increases portability. Additional miniaturization is achieved through an integrated heating element covering 24 parallel micro-reactors with a reaction volume of 0.14 µl each. The high level of parallelization allows the simultaneous analysis of 4 short tandem repeat (STR) loci and the amelogenin gender marker commonly included in forensic DNA analysis. A reference and crime scene sample can be analyzed simultaneously for direct comparison. Importantly, by using industry-standard semiconductor manufacturing processes, mass manufacturability can be guaranteed. Following assay design and optimization, complete 5-loci profiles could be robustly generated on-chip that are on par with those obtained using conventional benchtop real-time PCR thermal cyclers. Together, our results are an important step towards the development of commercial, mass-produced, portable devices for on-site testing in forensic DNA analysis.

## Introduction

Human DNA fingerprinting is currently almost exclusively performed based on short tandem repeat (STR) region identification^1,2^. Because of the highly polymorphic nature of these repetitive sequences, very distinctive profiles can be generated from minute amounts of DNA allowing discrimination of different individuals. Hence, a global consensus to use specific STR loci was agreed upon, allowing straightforward comparison of profiles in databases around the world^3,4^. The gold standard method for generating STR profiles was optimized about two decades ago and consists of a combination of multiplex polymerase chain reaction (PCR) and a subsequent fragment sizing using capillary electrophoresis (CE)^5^. Although delivering excellent results, this method of STR profiling does have some drawbacks. This process, which typically takes place in a specialized forensic laboratory, is labor-intensive, often taking several days to complete^6^. Several criminal investigations would benefit tremendously from having DNA profile information available within the first hour after discovery of the crime scene, the so-called golden hour^7^. This information could be used to focus the investigation on certain suspects and exclude innocent suspects at an early stage. An initial step would be to overcome some of the logistical steps of a traditional DNA fingerprinting analysis and perform a rapid on-site DNA screening. Lab-on-a-chip (LoC) technology could address some of the limitations that hamper the conventional STR typing process and thereby provide fast, correct information ad hoc^8^. LoCs typically have a significantly reduced footprint compared to benchtop tools making the analysis portable and allowing on-site testing. Furthermore, because of the small reaction volumes, LoCs typically have limited reagent consumption. Smaller devices which contain smaller amounts of liquids also have a lower thermal mass, which is of great importance when performing fast PCR amplification^9,10^. By using silicon as the fabrication substrate, the thermal inertia is reduced even further, resulting in modest power consumption and the possibility of integration into portable devices. Crucially, the use of industry-standard silicon semiconductor manufacturing processes for the fabrication of silicon LoCs guarantees mass manufacturability, which is an important consideration for future commercial development of single-use, disposable DNA fingerprinting chips. The level of integration that can be achieved using these fabrication techniques enables the production of chips that comply with the sample-in answer-out principle. Therefore the need for any manual intervention during the analysis process can be largely eliminated. Numerous research groups from both academia and industry have been working towards integrated systems for STR profile generation^11,12^. The PCR LoC based solutions described in the literature are mainly focused towards point of care applications and are limited in terms of reaction multiplexing^13,14^. Furthermore, many of these LoCs rely on-chip reagent storage, which is challenging and results in an increased device fabrication complexity^15,16^. In this paper a forensic LoC was designed, fabricated and tested capable of performing multiple PCR amplifications and STR detection without the need to perform CE analysis on-chip. Although STR profiling using CE-based size separation has been the gold standard for traditional DNA fingerprinting analysis, attempts at miniaturization of CE by on-chip implementation have been unable to deliver the single-basepair resolution that is required for forensic applications. Hence, alternative STR typing methods were developed and integrated on-chip. The HyBeacon technology used in LGC’s ParaDNA system holds particular promise to be integrated in a silicon LoC^17^. This HyBeacon technology relies on the determination of the melting temperature (T_m_) of a duplex formed by a HyBeacon probe and an STR-amplicon. The fluorescently labeled HyBeacon probe, complementary to a section of the repeat region, will dissociate at a characteristic temperature, the melting temperature, leading to a decrease in fluorescence. A longer STR region can hybridize with the probe over a longer distance resulting in a stronger interaction, requiring more energy (a higher temperature) to dissociate the complex. Therefore, the T_m_ of the probe-template duplex can be used as an indicator of the number of repeat regions. The HyBeacon assays used in this study are based on previous work by French *et al*. and Gale *et al*.^18,19^ with minor modifications. Assays for STR loci D16S539, D18S51, D8S1179, TH01 and the amelogenin sex marker were tested. The gender identification assay, which is based on the amelogenin gene, is a novel design. In order to test all five loci simultaneously, a minimum of 11 cavities is required. Multiple cavities per locus are needed to allow detection of the entire range of alleles. When long STRs are analyzed, a length difference of 1 STR (typically 4 nucleotides), only has a relatively small impact on the T_m_, making it difficult to distinguish the T_m_s of STRs with varying lengths. This problem is resolved by the use of non-fluorescent blocker sequences that block a predetermined number of STR repeats. This limits the number of repeats available for probe hybridization and increases the difference in T_m_ between unblocked STRs of varying length. Supplementary Figs 1 and 2 schematically show the blocking mechanism of the non-fluorescent blockers and explains the need for multiple blockers per locus. To detect the full range of common alleles, up to three blocker sequences per locus are required. Therefore, a LoC capable of simultaneously amplifying and melting more than 11 reactions had to be designed and tested. To allow a reference and crime scene sample to be analyzed simultaneously a LoC capable of analyzing 24 assays in parallel was designed. The miniature chip holds 24 small, reaction cavities for parallel testing and an integrated aluminum heating system. Temperature monitoring was accomplished by a resistance temperature detector (RTD) deposited directly on the silicon surface. The performance of this novel LoC to generate STR profiles using HyBeacon technology was evaluated. All modified HyBeacon assays were tested using conventional PCR amplification and melting analysis in a benchtop instrument before being transferred on-chip. Next, on-chip temperature uniformity during heating was assessed over all cavities. Finally, complete STR profiles of three reference samples were generated on-chip and compared to off-chip results.

## Materials and Methods

### Chip design and manufacturing

**Figure 1:**
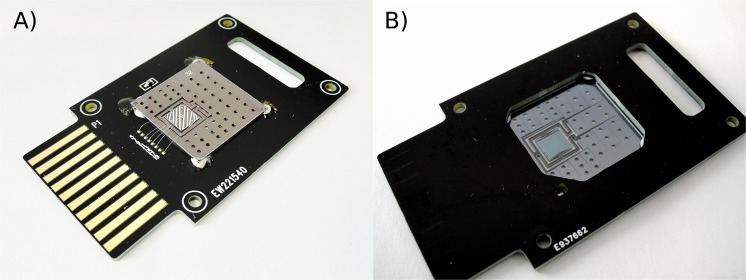
(**A**) Top view showing the complete chip including integrated heater, air trenches surrounding the reaction cavity, 24 inlets and outlets and the PCB. The integrated heater and resistance temperature detector are wire-bonded to the 10-pin connector. (**B**) Backside of the mounted chip showing the microfluidic channels connecting the different access holes with the respective reaction cavities. The backside is sealed with a Pyrex glass cover allowing fluorescence detection.

A LoC was designed with 24 parallel reaction cavities, which allows simultaneous PCR amplification of 24 independent reaction mixtures. Figure 1A shows the topside of the 24 reaction chamber LoC with the different access holes and the integrated aluminum heating element. The backside is shown in Fig. 1B, displaying the microfluidic channels connecting the inlet and outlet holes with the reaction cavities in the thermally isolated zone of the chip. The microfluidic structures were etched in a 0.4 mm thick silicon wafer that was subsequently diced into 4 cm^2^ individual chips. First, the reaction cavities and associated microfluidic channels were created on the front side of the silicon substrate by 0.25 mm deep reactive dry etching and subsequently sealed by anodic bonding of Pyrex. The air-trenches for thermal isolation of the reaction cavities, as well as the access ports for fluidics were created by subsequent backside dry etching up to the Pyrex-silicon interface. Each of the 24 reaction cavities has a volume of 0.14 µl with individual inlet and outlet channels connecting the access holes. The chip fabrication process is based on previously published work^10,20–22^. Supplementary Fig. 3 shows a schematic representation of the chip structures etched in the silicon substrate. To supply heat to the micro-reactors we used a PCR chip equipped with an integrated Joule heater consisting of a 200 nm aluminum thin film resistor patterned similar to Barman *et al*.^23^. To accommodate the 24 PCR cavities, a 5 mm by 5 mm heated and thermally isolated zone was designed. This included an adjusted routing of the microfluidic channels and optimized thermal air trench design to connect the 24 cavities with their respective inlet and outlet holes. Prior to fabrication, the thermal characteristics of the LoC were evaluated by numerical modeling. The largest temperature differences during heating are expected at the inlet and outlet regions. At these points the PCR cavities are connected to the rest of the chip through silicon beams which conduct heat to the rest of the chip. Actual temperature uniformity, achieved using the integrated heating system, was tested by performing a melt curve analysis of a calibration melt solution (1 X EvaGreen (Biotium), 50 ng/µl NoLimits 300 bp DNA Fragment (Thermo Fisher), 1 X DNA Taq polymerase Buffer (Thermo Fisher)). A uniform spatial reduction of the fluorescence level throughout the PCR reactor during heating is the result of uniform melting and hence indicates uniform heat transfer. A full description of the heating uniformity test as well as the development of the integrated heater is described by Barman *et al*.^23^. The small mass and the minute reaction volume of the chip result in a low thermal mass enabling rapid and efficient heat transfer which allows for fast thermal cycling. Cooling relies on natural convection and is the main factor limiting the speed of this thermal cycling. The lack of an active cooling system is intentional to keep the design and fabrication simple. On-line temperature monitoring is accomplished by a calibrated resistance temperature detector (RTD) placed alongside the heater at the center of the reaction chamber on the silicon surface. The chip is bound to a printed circuit board (PCB) using a thermally non-conductive epoxy. The heater and temperature sensor are connected to the PCB pins using 25 µm aluminum wires. A standard 10-pin socket connects the PCB with a temperature controller. The reagents are loaded on the chip using a 100 µl syringe (Hamilton, USA) and a Legato 180 syringe-pump (KD Scientific, USA) coupled to a capillary (ID 182.5 µm) (Polymicro technologies, USA) pumping the PCR reagents and an FC40 oil plug (F9755, Sigma Aldrich St Louis USA) at a flowrate of 8 µl/min. Injecting the oil plug prior to the PCR reagents prevents the formation of air bubbles in the reaction cavity during chip loading.

### Assays

Probe, blocker and primer sequences as well as the respective concentrations used in each assay (D16S539, D18S51, D8S1179, TH01) can be found in Supplementary Tables 1–4. All assay compositions are based on previous published work by French *et al*. and Gale *et al*.^18,19^. To promote the binding of the probe to its complementary strand, all amplification reactions are asymmetric PCRs. An asymmetric PCR amplification is achieved by using a 10 to 1 ratio of both primers. The 10-fold excess of one of both primers results in amplification of mainly the strand complementary to the probe. These HyBeacon probes consist of an oligonucleotide sequence specific for the STR target, one or several internal FAM fluorophore dT-analogues and a 3′ phosphate cap to prevent PCR extension. HyBeacon probes demonstrate increased fluorescence on hybridization with target DNA due to a conformational change of the fluorophore’s position, resulting in the fluorophore being positioned farther from adjacent quenching nucleotides^24^. Different blocker sequences with varying lengths are added in parallel to different reactions to block a fixed portion of the STR sequence of the target DNA from the probe. This ensures that a wide range of STRs can be analyzed using the same probe while maintaining considerable separation of the melting temperatures of consecutive repeats. The mechanism of probe and blocker binding is explained in Supplementary Figs 1 and 2 elaborately described by French *et al*.^19^. An assay for sex determination using the amelogenin gene was developed based on the same quenching and de-quenching principle as the other HyBeacon probes. The amelogenin assay consists of primers and a probe, but no blocker sequence was needed as only two alleles have to be discriminated from each other. The probe consists of a nineteen nucleotide long sequence and a single FAM fluorophore linked to an internal dT-analogue. Unlike the previously described HyBeacon probes, where the difference in melting temperature is based on a different number of nucleotides binding with the probe, the difference in melting temperature is based on the probe-target duplex stability. The probe’s sequence is perfectly complementary with the amelogenin gene on the X chromosome, whereas a dual SNP on the Y chromosome induces a mismatch and an associated duplex destabilization. The primer and probe sequences, as well as the respective concentrations used in the amelogenin assay can be found in Supplementary Table 5.

### Amplification

Each of the previously described assays were first tested using a conventional LightCycler 480 instrument (Roche, Switzerland) to verify their efficacy and to produce a benchmark melting temperature for a broad range of alleles. All alleles with prevalence larger than 0.6% according to the *popSTR* Europe database (spsmart.cesga.es/popstr.php) were analyzed in triplicates. Samples were obtained from the GEDNAP (German DNA Profiling, www.gednap.org) proficiency tests. All amplifications were conducted in a 10 µl volume, containing 1 X Qiagen PCR buffer, 1 U HotStarTaq polymerase (Qiagen, Crawley, UK), 250 µM of each dNTP (Thermo Fisher Scientific, Waltham, USA) and the specific amounts of primers, probe and blockers (see Supplementary Tables 1–5). Amplifications were performed asymmetrically to generate an excess of target strand assuring that probe hybridization is favored over reannealing of the amplified sequences, as already mentioned. Amplification was initiated with an initial denaturation for 15 min at 95 °C to activate the HotStarTaq polymerase, followed by 45 cycles consisting of denaturation (95 °C, 15 s), annealing (55 °C, 30 s) and extension (72 °C, 30 s). Melting analysis was performed by heating the PCR product from 40 °C to 65 °C at a rate of 0.1 °C/s. This results in a total analysis time below 70 minutes. During this melting the fluorescence intensity was continuously monitored and melt peaks were constructed by plotting the negative first derivative of the fluorescence intensity with respect to the temperature. Furthermore, melting analysis was performed on three reference DNA samples (9948, 9947 and 2800) (Promega, USA).

These three reference DNA samples were also subjected to on-chip analysis using identical thermal cycling and melting parameters as described for the off-chip experiments. Reference STR profiles of these samples were obtained using conventional PCR-CE analysis and can be found in Supplementary Table 6. Although the on-chip reaction volume is reduced over 70 times (10 µl vs 0.14 µl) compared to the initial off-chip tests, identical reagent concentrations were used as described above. Some alterations were made to counteract the possible effects of the silicon surface on the PCR efficiency. More specifically, the polymerase concentration was increased to 5 U/µl and Bovine Serum Albumin (BSA) was added to the master mix to passivate the silicon surface^25^. On-chip fluorescent readout was performed using a setup consisting of a mercury lamp (X-cite *exacte*, Excelitas, Canada) with appropriate filter set for FAM fluorophore detection (89016 Chroma Vermont, USA) and an inverted microscope (IX73 Olympus, Japan) combined with an Orca flash 4.0 CMOS camera (Hamamatsu, Japan). The thermal cycling program and temperature monitoring were controlled by a custom LabVIEW® script linked to a thermal controller unit (3504 Eurotherm Ashburn, USA) coupled to a 7 V DC power supply unit.

## Results

### Off-chip

#### Off-chip HyBeacon assay testing

Prior to profile generation using the LoC, all assays were tested on the LightCycler 480. This off-chip testing of each assay allowed validation of the slightly modified HyBeacon assays. All alleles of all loci were tested in triplicates using the respective blockers. Results of this off-chip testing can be found in Supplementary Tables 7–11. On average, the obtained T_m_ is 1.37 °C (±0.97 C) higher compared to previously published data by Gale *et al*. and French *et al*.^18,19^. Guidelines for the identification of the alleles were defined using these results. These results can be used to set up temperature ranges for each allele. When a T_m_ of an unknown sample lies within one of these ranges, the according allele is assigned to the investigated sample.

#### Off-chip STR profiling

The melting temperatures of the forensic reference Promega samples 2800, 9947 and 9948 were determined in triplicates (n = 3) using the LightCycler 480 instrument. Melting temperatures for all samples were generated using the full array of probe and blocker combinations. All T_m_ values are given in Table 1. Although some loci were homozygous, two distinct melting peaks can be observed when performing the melt analysis (for example D16Bl4–9948). These additional melting peaks originate when the probe binds before the blocker can, resulting in full probe hybridization. These full probe melting peaks are a known phenomenon and are not associated with an allele. They are therefore indicated by FP in Table 1 and not considered when composing the final STR profile. For each sample a complete STR profile could be generated based on the T_m_ values. These STR profiles were in perfect concordance with the reference STR profiles generated with PCR-CE. By performing these off-chip profiling experiments the efficacy of the slightly adjusted assays could be evaluated. Moreover, the performance of the novel sex identification assay was assessed as shown in Table 1.

**Table 1:** Overview of melting temperatures ± SD of reference samples 9947, 9948 and 2800 determined using the benchtop LightCycler 480 instrument and the called alleles.

Locus	Blocker	9947		9948		2800	
		Tm (°C) ± SD(°C)	Observed alleles	Tm (°C) ± SD(°C)	Observed alleles	Tm (°C) ± SD(°C)	Observed alleles
D16S539	**Bl 4**	**56**.**2** ± 0.1	**11**	**56.1** ± 0.2	**11**	**48**.**0** ± 0.4 & **58**.**2** ± 0.2	**9: 11+**
	**Bl 6**	**48**.**5** ± 0.3 & **52**.**5** ± 0.4	**11: 12**	**49.1** ± 0.7 & **57.0** ± 0.6	**11: FP**	**56**.**7** ± 0.4	**13**
TH01	**Bl 2**.**1**	**58**.**5** ± 0.3	**8+**	**46.40** ± 0.4 & **59.0 **± 0.1	**6: 8+**	**45**.**6** ± 0.4 & **5829** ± 0.2	**6: 8+**
	**Bl 3**.**3**	**51**.**4** ± 0.2–**58**.**7** ± 0.5	**8: 9**.**3**	**58.9** ± 0.4	**9**.**3**	**58**.**7** ± 0.3	**9**.**3**
D8S1179	**Bl 5**	**59**.**6** ± 0.7	**11+**	**59.60** ± 0.36	**11+**	**60**.**0** ± 0.4	**11+**
	**Bl 8**	**51**.**2** ± 0.4	**13**	**47.5** ± 0.5 & **51.3** ± 0.4	**12: 13**	**55**.**9** ± 0.4 & **58**.**2** ± 0.3	**14: 14+**
	**Bl 11**	**58**.**5** ± 0.2	**FP**	**59.0** ± 0.7	**FP**	**41**.**5** ± 0.8 & **48**.**4** ± 0.3	**14: 15**
D18S51	**Bl 7**	**62**.**0** ± 0.3	**14+**	**61.7** ± 0.5	**14+**	**61**.**9** ± 0.4	**14+**
	**Bl 10**	**53**.**5** ± 0.2 & **62**.**1** ± 0.2	**15: 17+**	**53.8** ± 0.3 & **62.6** ± 1.0	**15: 17+**	**57**.**8** ± 0.2 & **62**.**6** ± 0.3	**16: 17+**
	**Bl 14**	**54**.**5** ± 0.1 & **61**.**9** ± 0.2	**19: FP**	**48.9** ± 0.3 & **62.2** ± 0.7	**18: FP**	**49**.**0** ± 0.5 & **62**.**3** ± 0.2	**18: FP**
Amelogenin		**50**.**9 ± 0**.**8**	**X**	**51.0** ± 1.0 & **60.9** ± 0.5	**X: Y**	**51**.**5** ± 0.8 & **60**.**8** ± 1.0	**X: Y**

A plus (+) indicates the maximum detectable allele is achieved, FP indicates a full probe peak is measured.

### On-chip

#### Chip loading

Loading of the novel 24 cavity chips was performed using a syringe-pump coupled to a capillary allowing a constant injection flowrate. This constant flowrate prevents pressure surges and thereby air bubble formation. To further reduce air bubble trapping, each PCR cavity was flushed and loaded with FC40 oil (Fluorinert, 3 M) prior to loading the sample solution. This FC40 oil is more resilient to air bubble formation due to its low contact angle with silicon. Upon loading the hydrophilic sample- master mix solution, the FC40 oil plug is pushed out.

#### Temperature uniformity

**Figure 2:**
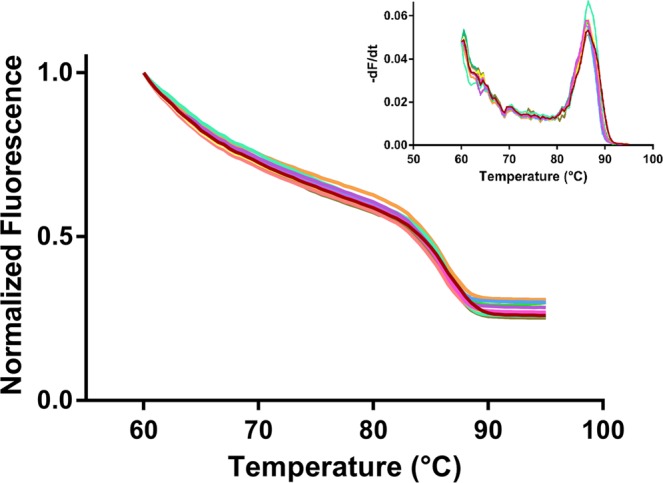
Melting curves of the 24 individual reaction cavities generated using the normalized fluorescence intensity of the NoLimits 300 bp DNA fragment melt solution. Melting peaks, in the insert, are presented as the first negative derivate of the melting curves. An average T_m_ of 86.31 (±0.25 °C) was observed with no differences observed between the inner and outer reaction cavities.

Numerical modeling allowed predicting the thermal properties of the novel 24-cavity chip design prior to fabrication. The reactor zone was divided into equally sized modular blocks of which 93.05% where within the set temperature ±0.3 °C. The thermal numerical modeling results can be found in Supplementary Figure 4. Thermal uniformity was tested to verify the numerical modeling data. This was performed by means of melting analysis of a calibration solution. Each reaction cavity was filled with the calibration dye and subjected to a melting analysis. The results of the thermal uniformity tests can be found in Fig. 2. The average observed T_m_ in the 24 cavities was 86.31 °C (±0.25 °C) with no significant differences observed between the inner and outer reaction cavities. The uniformity within each reaction cavity was also tested by measuring the fluorescence intensity of three discrete regions (top, middle bottom). No differences were found between each of the three regions. As a comparison, a melting analysis was also performed on the LightCycler 480 instrument using the same calibration dye. The resulting T_m_ was 89.24 °C (±0.32 °C), which is 2.93 °C (±0.33 °C) higher compared to the results obtained by on-chip melting analysis.

#### On-chip STR profiling

**Figure 3:**
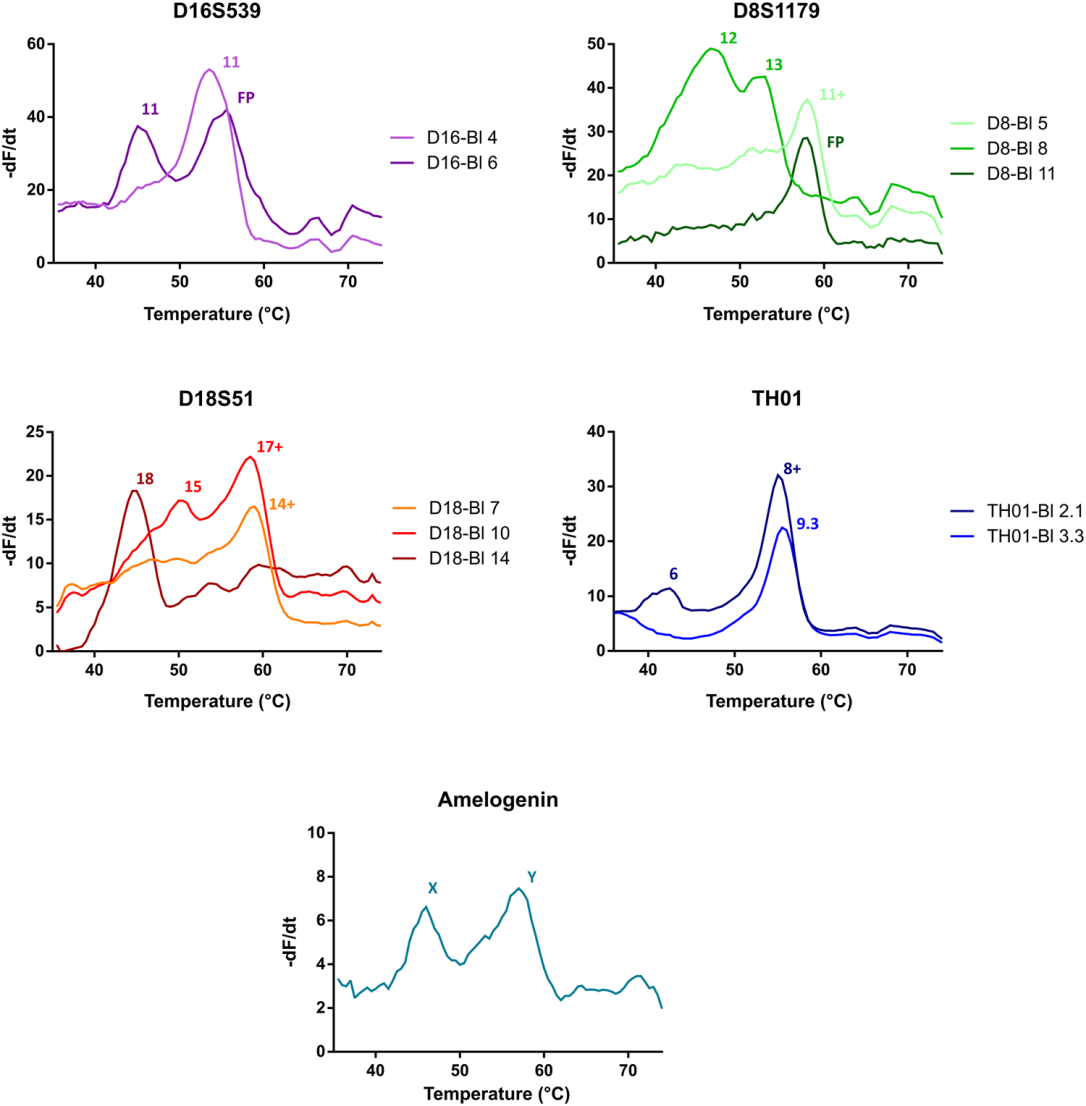
On-chip melting peaks profiles for the 9948 reference sample using the D16S539, D18S51, D8S1179, TH01 and Amelogenin HyBeacon probes. The alleles corresponding to the acquired melting temperatures are indicated above each respective melting peak. A plus (+) indicates the maximum detectable allele is achieved, FP indicates a full probe peak is measured.

STR profiling using the HyBeacon assays was performed on-chip for the 9947, 9948 and 2800 reference samples. The novel developed 24 cavity chips allow testing of the full array of probe and blocker combinations for all loci in parallel. Moreover, enough cavities are available to perform two analyses on the same chip, for example a suspect’s reference sample and crime scene sample. Figure 3 shows the on-chip generated melting peaks of the 4 investigated STR loci and the amelogenin marker for the 9948 sample. The melting temperatures of all blocker combinations for the 9947, 9948 and 2800 samples were determined in triplicates and can be found in Table 2. The potential of the amelogenin assay to determine the gender of the sample’s donor is shown in Supplementary Fig. 4. A 10 °C difference separates the melting peaks which correspond to the X and Y amelogenin alleles allowing for straightforward gender identification. It should be noted that, similar to the results with the calibration dye, all on-chip acquired T_m_ values are between 2.5 °C and 3 °C lower compared to the values obtained using the LightCycler 480 instrument. However, as the difference is constant over all analyses, the produced T_m_s hold value and can be readily used to generate STR profiles.

**Table 2:** Overview of T_m_ values of reference samples 9947, 9948 and 2800 as determined on a 24-cavity silicon chip.

Locus	Blocker	9947		9948		2800	
		T_m_ (°C) ± SD(°C)	Observed alleles	T_m_ (°C)_ ± _SD(°C)	Observed alleles	T_m_ (°C)_ ± _SD(°C)	Observed alleles
D16S539	**Bl 4**	**53**.**7** ± 0.8	**11**	**53**.**5** ± 0.7	**11**	**45**.**5** ± 0.9 & **53**.**7** ± 0.3	**9: 11+**
	**Bl 6**	**46**.**3** ± 0.5 & **51**.**16** ± 0.48	**11: 12**	**45**.**6** ± 0.3 & **55**.**5** ± 0.3	**11: FP**	**53**.**3** ± 0.6	**13**
TH01	**Bl 2**.**1**	**55**.**5** ± 0.4	**8+**	**42**.**0** ± 0.6 & **55**.**4** ± 0.9	**6: 8+**	**43**.**6** ± 1.8 & **56**.**9** ± 0.9	**6: 8+**
	**Bl 3**.**3**	**49**.**2** ± 1.1 & **56**.**3** ± 0.7	**8: 9**.**3**	**56**.**8** ± 0.4	**9**.**3**	**56**.**5** ± 0.6	**9**.**3**
D8S1179	**Bl 5**	**56**.**8** ± 0.6	**11+**	**57**.**2** ± 0.8	**11+**	**55**.**3** ± 0.6	**11+**
	**Bl 8**	**49**.**2** ± 0.7	**13**	**45**.**5** ± 1.0 & **49**.**5** ± 1.7	**12: 13**	**52**.**9** ± 0.6 & **56**.**2** ± 0.4	**14: 14+**
	**Bl 11**	**57**.**1** ± 0.3	**FP**	**57**.**3** ± 0.4	**FP**	**/**±(−) **& 45**.**6** ± 0.5	**14: 15**
D18S51	**Bl 7**	**58**.**2** ± 0.7	**14+**	**59**.**1** ± 0.8	**14+**	**58**.**6** ± 0.7	**14+**
	**Bl 10**	**50**.**3** ± 0.6 & **58**.**2** ± 0.6	**15: 17+**	**50**.**0** ± 0.5 & **59**.**1** ± 0.7	**15: 17+**	**54**.**7** ± 0.3 & **58**.**3** ± 0.7	**16: 17+**
	**Bl 14**	**50**.**3** ± 0.3 & **59**.**0** ± 0.8	**19: FP**	**45**.**3** ± 0.6 & **60**.**1** ± 0.5	**18: FP**	**44**.**8** ± 0.7 & **59**.**1** ± 0.3	**18: FP**
Amelogenin		**47**.**2** ± 0.5	**X**	**48**.**1** ± 0.9 & **58**.**8** ± 0.5	**X: Y**	**47**.**8** ± 1.1 & **58**.**4** ± 0.9	**X: Y**

The called alleles shown for each locus, a plus (+) indicates the maximum detectable allele is achieved, FP indicates a full probe peak is measured. This occurs when the blocker insufficiently blocks the target regions.

## Discussion

### Chip loading

Prior to the amplification and the analysis, all the assays were loaded in separate cavities. Substantial effort was invested in the prevention of air-bubble formation in the PCR cavities as trapped air will expand and contract during the heating and cooling cycles. These repetitive forces will drive the PCR solution out the heated zone resulting in failure of the amplification. By filling the PCR cavities with FC40 oil air-bubble formation is averted. Due to the low contact angle of the FC40 oil with silicon it simply fills the cavities without any spraying effects. The FC40 oil does not interfere with the amplification nor with the melting analysis^26,27^. Other factors influencing the likelihood of air-bubble formation can be dealt with at the design stage. Serpentine-like shaped cavities are favored, as this design helps to expel the air bubble once being formed.

### Off-chip assay development

Off-chip assay testing allows us to identify whether or not the small changes made to the chemistry of the probes and the blockers influenced the efficacy of the assay. Minor differences in T_m_, compared to what is reported in literature were observed. These minor discrepancies are attributed to small differences in both the master mix composition and the probe and blocker sequences. For example the probes used in this study did not contain a 5′ trimethoxystilbene (TMS) cap which has a stabilizing effect on the probe-target duplex due to stacking interactions^28,29^, thereby influencing melting temperature. Although the minimal modifications to the assays resulted in some minor T_m_ differences robust amplification and detection could be achieved. Besides altering some previously developed assays for the STR loci, we designed a completely novel assay for the amelogenin locus. Unlike the STR assays the amelogenin assay did not contain blocker sequences. Discrimination between the XX ad XY genotypes solely relied on probe complementarity. The amelogenin probe was designed to be fully complementary with the Y allele, while having a 2 nucleotide mismatch with the X allele. Upon annealing of the probe with the X allele, the 2 nucleotide mismatch results in duplex destabilization. When performing a melting analysis this destabilized duplex requires less energy to melt compared to the fully complementary Y allele. The resulting T_m_ difference of 10 °C allows for unbiased gender identification.

### On-chip assay testing

Upon transferring the assay on-chip it was obvious that the PCR efficiency was lower compared to the off-chip experiments. The increased surface-area-to-volume ratio and the tendency of silicon to adhere biological macromolecules, such as polymerases, led to a reduced amount of Taq polymerase available for amplification. By flushing the chip with BSA prior to PCR reagent loading, the silicon interface is lined with BSA which largely prevents the adsorption of the Taq polymerase^25,30^. This passivation increased the PCR amplification efficiency considerably. Compared to the STR assays the amelogenin assay typically showcased a low intensity of the melting peaks when analyzed on-chip. Adding a second fluorophore to the amelogenin probe could probably overcome this issue. The second fluorophore, positioned at the optimum interval of 5 to 7 nucleotides and preferably next to a guanine or cytosine nucleotide, could act as an additional fluorescent quencher producing more intense and sharper melting peaks^19,24^. This rationale is supported by the D16S539 assay in which the probe contains three fluorophores. Throughout all analyses the D16S539 assay displayed the largest drop in fluorescence and strongest melting peaks being straightforward to interpret. The observed discrepancy of 2.5 °C to 3 °C between the detected melting temperature when performed either on-chip or the LightCycler 480 instrument, was consistent over all analyses. This observed discrepancy between both analysis methods is most likely due to the differences in thermal set-up and volume. Experiments using different ramp rates or geometrical set-ups resulting in different off-sets have been reported before^31^. Of vital importance to the application described here, is the high reproducibility of the observed melting temperatures on both systems. This allows both to be used readily to generate STR profiles.

An important consideration to make concerning the on-chip analysis is the fact that the sample has to be divided over several cavities in order to generate a complete STR profile. Therefore, if low input samples are examined, they might become too diluted to result in good and balanced amplification. This technology should therefore be seen primarily as an initial screening assay for testing high quality and quantity samples such as saliva swabs or extracted blood^32,33^.

### Future perspectives

The HyBeacon technology has already proven its value in LGC’s ParaDNA system. This system allows police investigators to produce database compatible DNA profiles close to or at the site of sampling within an hour^27^. Several validation studies were performed allowing the system to transit form proof-of-concept to an operationally ready device^13,28^. However, its introduction into general routine use has been partially hampered by the system’s dimensions, weight and cost. By applying the HyBeacon technology on the herein developed 24 cavity µPCR chip, these issues are partially surmounted. The use of small and lightweight chips makes on-site analysis conceivable. Moreover, by solely relying on standard semi-conductor fabrication processes and methods used in the CMOS industry, low cost and high volume production can be guaranteed. An additional advantage of using silicon is the fact that it holds potential to speed up the thermal cycling of PCR amplification, which has recently been demonstrated^34,35^. Even though some great leaps towards cheap, on-site human DNA fingerprinting have been made, further developments are still needed to achieve a similar level of integration as showcased by the ParaDNA system. An easy to use sampling device, as well as a sample introduction system have to be developed to allow non-trained operators to load a sample without the use of any laboratory equipment. In addition to a user friendly loading mechanism, integrated chips should include a sample preparation and a DNA extraction step. Some initial work with positive results has already been performed in which the integrated heater completes a thermal cell lysis to release the DNA. Many different on-chip extraction techniques have already been explored. The majority of methods use solid-phase based sample preparation techniques; which utilize the differential binding capacities of silica^36–38^. Furthermore, to limit the operator’s hands-on time, pre-loading of all necessary reagents is an absolute necessity. As anodic bonding of the Pyrex glass with the silicon chip typically requires high temperatures (250–400 °C), it cannot be performed after the thermolabile reagents are deposited. Alternatively, the reagents could be stored in a separate cartridge in which a silicon chip is embedded. This would circumvent the technical challenges involved with maintaining reagent stability during the different chip production processes. Finally the optical setup for a fluorescent readout should be miniaturized as well using integrated photonics or a small detection system^39^.

## Conclusion

In this work the potential of an integrated silicon µPCR chip for the amplification of STR loci and detection of the alleles using a HyBeacon assays was demonstrated. A µPCR chip capable of performing 24 analyses in parallel is designed and produced relying solely on standard semi-conductor fabrication processes. The potential of this µPCR chip to perform on-site DNA-fingerprinting was demonstrated by repeatedly and correctly producing STR profiles of three reference samples. In addition to the previously developed D16S539, D18S51, D8S1179 and TH01 assays the generated STR profiles also contain a sex identifier. A novel amelogenin gender determination assay was developed and optimized for on-chip analysis. This work is an important step towards the development of a handheld forensic analysis tool for on-site DNA fingerprinting.

## Supplementary information

Supplementary information
