# Supplementary material for: Silicon µPCR Chip for Forensic STR Profiling with Hybeacon Probe Melting Curves
Source: Sci Rep. 2019 May 14;9:7341. doi: 10.1038/s41598-019-43946-5 (PMC6517373; doi:10.1038/s41598-019-43946-5)
Supplement: Supplementary file 1 — Supplementary information [file 41598_2019_43946_MOESM1_ESM.pdf]

SILICON µPCR CHIP FOR FORENSIC STR PROFILING WITH HYBEACON PROBE MELTING CURVES

AUTHORS

Senne Cornelis[1,2], Olivier Tytgat[1,2], Maarten Fauvart[2], Yannick Gansemans[1], Ann-Sophie Vander Plaetsen[1], Rodrigo S Wiederkehr[2], Dieter Deforce[1,#,*], Filip Van Nieuwerburgh[1,#], Tim Stakenborg[2,#]

[1] Laboratory of Pharmaceutical Biotechnology, Ghent University, 9000 Gent, Belgium
[2] Department of Life Science Technologies, Imec, 3001 Leuven, Belgium.
# Contributed equally

* Corresponding author at: Laboratory of Pharmaceutical Biotechnology, Faculty of Pharmaceutical Sciences, Ghent University, Ottergemsesteenweg 460, 9000 Gent, Belgium.
Tel: +32 (0)9 264 80 48

DECLARATION OF INTEREST

None

EMAIL ADDRESSES

Senne Cornelis:	Senne.Cornelis@UGent.be,
Olivier Tytgat:	Ojtytgat.Tytgat@UGent.be,
Maarten Fauvart:	Maarten.Fauvart@imec.be,
Yannick Gansemans:	Yannick.Gansemans@UGent.be,
Ann-Sophie Vander Plaetsen:	AnnSophie.VanderPlaetsen@UGent.be,
Rodrigo Sergio Wiederkehr:	Rodrigo.Sergio.Wiederkehr@imec.be,
Dieter Deforce:	Dieter.Deforce@UGent.be,
Tim Stakenborg:	Tim.Stakenborg@imec.be,
Filip Van Nieuwerburgh:	Filip.VanNieuwerburgh@UGent.be

SUPPLEMENTARY INFORMATION

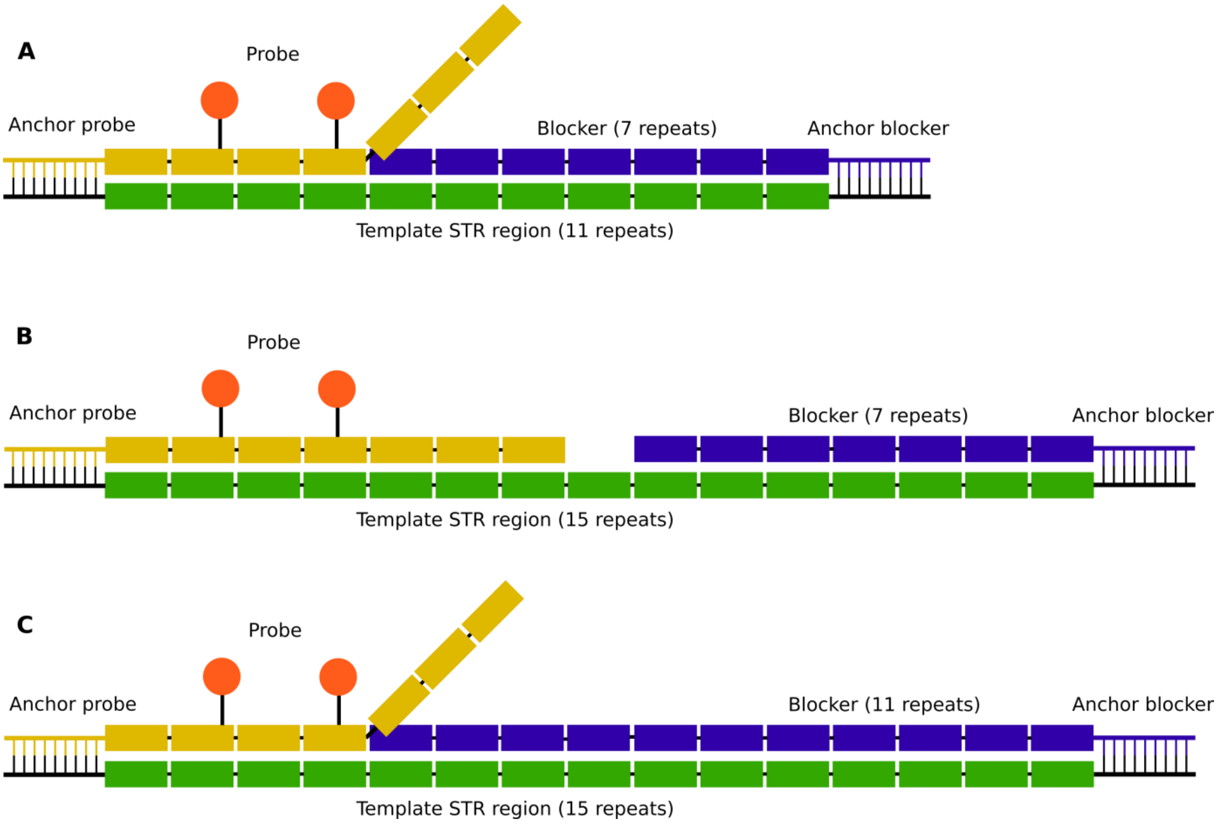

Supplementary Figure 1: Schematic overview of the HyBeacon system with a fluorescent HyBeacon probe and non-fluorescent blocker oligonucleotide. Probe, blocker and target sequence repeats are represented by yellow, blue and green boxes, respectively. Both probe and blocker oligonucleotides possess anchor regions that hybridize to non-repetitive flanking sequences to prevent slippage [[19]]. **A)** STR target region comprising of 11 repeat units partially blocked by a 7 repeat blocker and bound to the probe. **B)** STR region comprising of 15 repeat units in combination with a 7 repeat blocker results in full probe hybridization which makes discrimination with a 14 repeat STR region unfeasible. **C)** A blocker of 11 repeat blocks a larger fraction of the 15 repeat target sequence which allows partial probe hybridization and identification of the 15 STR region.

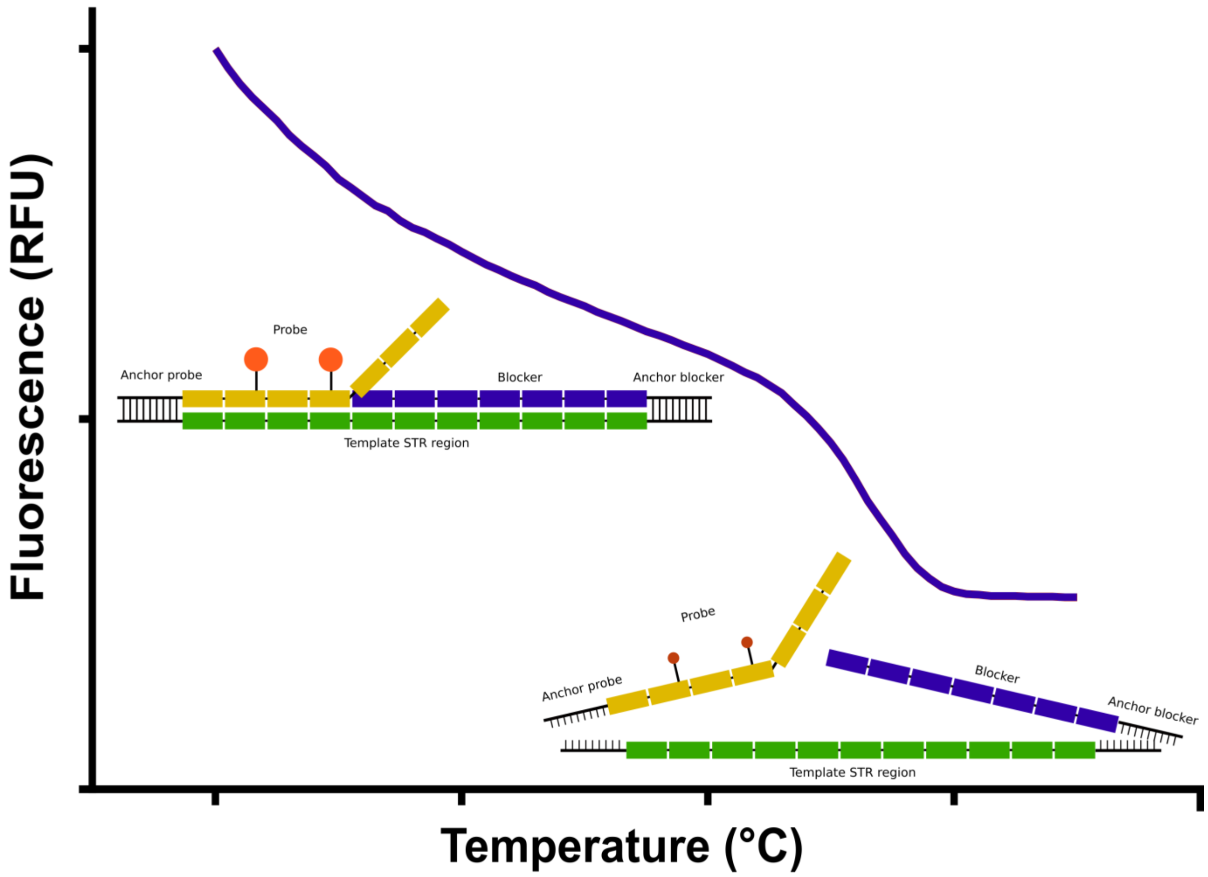

Supplementary Figure 2: Schematic overview of the working mechanism of a fluorescent HyBeacon probe and blocker oligonucleotide. Probe, blocker and target sequence repeats are represented by yellow, blue and green boxes, respectively. Both probe and blocker oligonucleotides possess anchor regions that hybridize to non-repetitive flanking sequences to prevent slippage [[19]]. In hybridized state no quenching of the probes' fluorophores is experienced and high fluorescence intensity is observed. Upon heating denaturation of the probe is favored resulting in a quenching effect which reduces the fluorescence intensity.

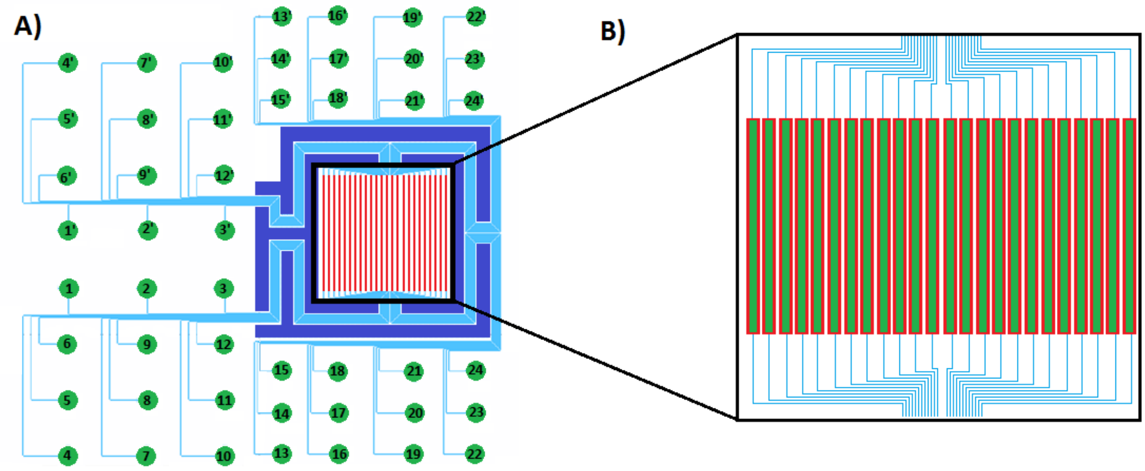

Supplementary Figure 3: A) Schematic overview of the 24 reaction cavity chip with, inlet and outlet holes (green), microfluidic channels (light blue) from and to the reaction cavities (red) and the air trenches etched in silicon (dark blue). The inlet and outlet holes are numbered from 1-24 according to the position of reaction chambers from left to right. **B)** A close-up of the 24 parallel reaction cavities and the microfluidic inlet and outlet channels leading to them.

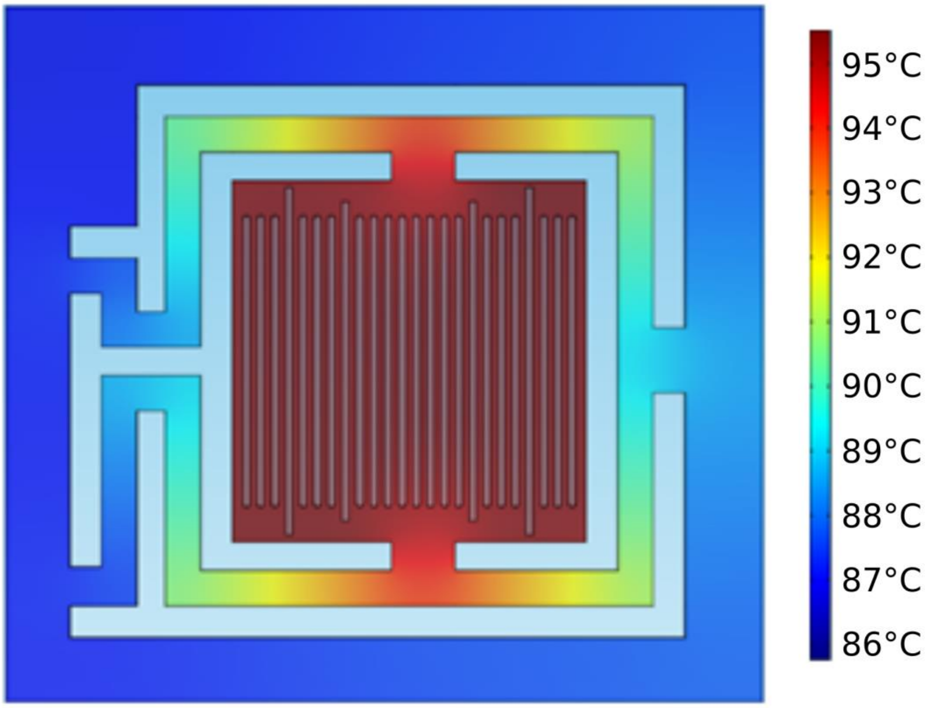

Supplementary Figure 4: Numerical modeling was performed to estimate the thermal characteristics of the novel chip design accommodating 24 PCR cavities with a total heated zone of 5 mm by 5 mm. 93.05 % of the heated zone was within± 0.3°C of the set temperature.

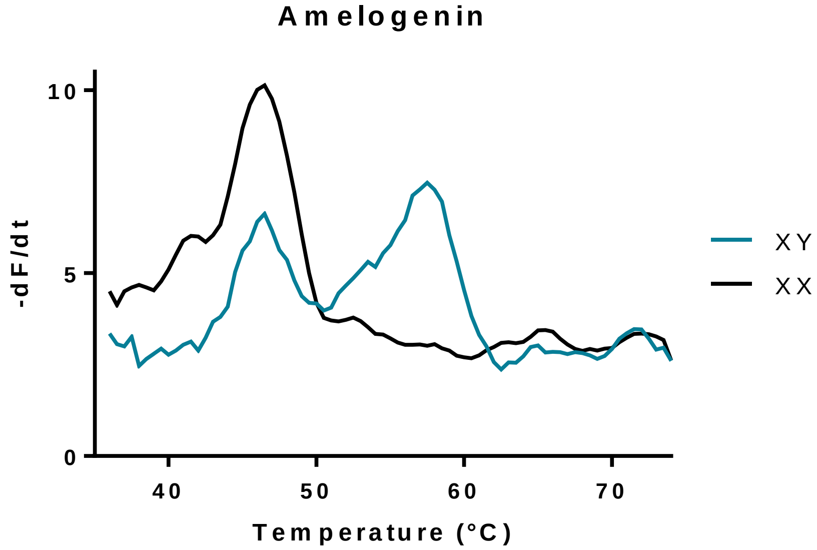

Supplementary Figure 5: Melting peaks generated using the amelogenin assay of a male (XY) and female (XX) sample.

**Name (D18S51)**	**Sequence**	**Concentration**
Forward primer	TGCCACTGCACTTCACTCTGA	1 µM
Reverse primer	GTGTGGAGATGTCTTACAATAACAGTTG	0.1 µM
HyBeacon probe	TTCTTTCTTTCTTTCTTTCTTTCTT**F**CTTTC**F**GAGACP	0.150 µM
D18 Blocker 7	CTTTCCTCTCTCTTT$(TTCT)_7$P	0.225 µM
D18 Blocker 10	CTTTCCTCTCTCTTT$(TTCT)_{10}$P	0.225 µM
D18 Blocker 14	CTTTCCTCTCTCTTT$(TTCT)_{14}$P	0.225 µM

<u>Supplementary Table 1</u>: Sequences of Probes, Primers and Blockers of the D18S51 locus assay and their respective concentrations. F: Internal fluorescein dT, P: 3' Phosphate cap.

**Name (D8S1179)**	**Sequence**	**Concentration**
Forward primer	CGGCCTGGCAACTTATATGT	0.1 µM
Reverse primer	GCCTTAATTTATTTACCTATCCTGTAGA	1 µM
HyBeacon probe	TCTATCTATCTATCTATCTATCTATC**F**ATCTA**F**TCCCCP	0.075 µM
D8 Blocker 5	GTATTTCATGTGTACATTCGTA$(TCTA)_5$P	0.375 µM
D8 Blocker 8	GTGTACATTCGTA$(TCTA)_8$P	0.375 µM
D8 Blocker 11	GTGTACATTCGTA$(TCTA)_{11}$P	0.375 µM

<u>Supplementary Table 2:</u> Sequences of Probes, Primers and Blockers of the D8S1179 locus assay and their respective concentrations. F: Internal fluorescein dT, P: 3' Phosphate cap.

**Name (TH01)**	**Sequence**	**Concentration**
Forward primer	GGCTTCCGAGTGCAGGTCA	0.1 µM
Reverse primer	GGTGATTCCCATTGGCCTG	1 µM
HyBeacon probe	TGG**F**GAATGAA**F**GAATGAATGAATGAATGAP	0.075 µM
TH01 Blocker 3.3	ATGAATGAATGAATGAGGGAAATAAGGGP	0.375 µM
TH01 Blocker 2.1	GAATGAATGAGGGAAATAAGGGAGGAACP	0.375 µM

<u>Supplementary Table 3:</u> Sequences of Probes, Primers and Blockers of the TH01 locus assay and their respective concentrations. F: Internal fluorescein dT, P: 3' Phosphate cap.

**Name (D16S539)**	**Sequence**	**Concentration**
Forward primer / Blocker 4	GCGGC$(TATC)_4$CACCTGTCTGTCTGTCTGTA-HEG-GATCCCAAGCTCTTCCTCTT	1 µM
Forward primer / Blocker 6	GCGGC$(TATC)_6$CACCTGTCTGTCTGTCTGTA-HEG-GATCCCAAGCTCTTCCTCTT	1 µM
Reverse primer	ACGTTTGTGTGTGCATCTGTAAGCATGTATC	0.1 µM
HyBeacon probe	TATCTATC**F**TCTATC**F**ATCTATC**F**ATCTATCGCCGCP	0.075 µM

<u>Supplementary Table 4:</u> Sequences of Probes, Primers and Blockers of the D16S539 locus assay and their respective concentrations. F: Internal fluorescein dT, P: 3' Phosphate cap, HEG: Hexaethylene glycol spacer.

**Name (Amelogenin)**	**Sequence**	**Concentration**
Forward primer	CCCTGGGCTCTGTAAAGAA	1 µM
Reverse primer	ATCAGAGCTTAAACTGGGAAGCTG	0.1 µM
HyBeacon probe	TCAAGTGG**F**CCCAATTTTACAGTTP	0.075 µM

<u>Supplementary Table 5:</u> Sequences of Probes, Primers and Blockers of the Amelogenine locus assay and their respective concentrations. F: Internal fluorescein dT, P: 3' Phosphate cap

	9947	9948	2800
D8S1179	13 : 13	12 : 13	14 : 15
D16S539	11 : 12	11 : 11	9 : 13
D18S51	15 : 19	15 : 18	16 : 18
TH01	8 : 9.3	6 : 9.3	6 : 9.3
Amelogenin	X : X	X : Y	X : Y

Supplementary Table 7.6: STR profiles based on the examined loci of three reference samples (9947, 9948 and 2800)

**D18S51**	**Allele**	**Tm 1 (°C)**	**Tm 2 (°C)**	**Tm 3 (°C)**	**Average Tm (°C)**	**SD**
Blocker 7	11	48,70	47,77	48,19	48,22	0,47
	12	54,20	53,01	54,47	53,89	0,78
	13	58,37	57,87	58,53	58,26	0,34
	14	61,20	62,24	61,03	61,49	0,66
	14+	62,75	63,10	62,79	62,88	0,19
Blocker 10	14	47,36	47,97	48,29	47,87	0,47
	15	54,14	54,33	54,80	54,42	0,34
	16	57,52	58,46	58,67	58,22	0,61
	17	61,23	61,25	62,30	61,59	0,61
	17+	62,43	62,72	63,35	62,83	0,47
Blocker 14	18	49,22	48,62	48,55	48,80	0,37
	19	55,62	54,62	54,51	54,92	0,61
	20	59,34	58,37	58,45	58,72	0,54
	21+	64,27	63,00	62,98	63,42	0,74

Supplementary Table 7: Melting temperatures of different alleles of the D18S51 locus using respectively the BL7, BL10 and BL14 blocker

**D8S1179**	**Allele**	**Tm 1 (°C)**	**Tm 2 (°C)**	**Tm 3 (°C)**	**Average Tm (°C)**	**SD**
Blocker **5**	8	41,32	40,81	39,91	40,68	0,71
	9	48,12	47,50	47,66	47,76	0,32
	10	51,84	52,65	52,10	52,20	0,41
	11	55,78	55,85	54,86	55,50	0,55
	11+	60,43	60,26	59,10	59,93	0,72
Blocker **8**	11	39,56	38,44	39,83	39,28	0,74
	12	46,63	48,27	47,18	47,36	0,83
	13	51,61	51,00	50,93	51,18	0,37
	14	54,00	55,72	54,94	54,89	0,86
	14+	57,43	56,50	57,65	57,19	0,61
Blocker **11**	14	39,14	39,60	39,86	39,53	0,36
	15	46,28	46,43	46,66	46,46	0,19
	16	50,41	50,93	51,26	50,87	0,43

Supplementary Table 8: Melting temperatures of different alleles of the D8S1179 locus using respectively the Bl5, Bl8 and Bl11 blocker

**TH01**	**Allele**	**Tm 1 (°C)**	**Tm 2 (°C)**	**Tm 3 (°C)**	**Average Tm (°C)**	**SD**
Blocker **2.1**	6	46,57	46,36	46,27	46,40	0,15
	7	52,44	52,25	52,60	52,27	0,24
	8+	58,63	58,46	58,42	58,50	0,11
Blocker **3.3**	8	50,76	51,46	51,84	51,35	0,55
	9	54,86	55,7	55,83	55,46	0,53
	9.3	58,45	58,74	58,94	58,71	0,25
	10	58,16	58,52	58,82	58,50	0,33

<u>Supplementary Table 9:</u> Melting temperatures of different alleles of the DTH01 locus using respectively the Bl2.1 and Bl3.3 blocker

**D16S539**	**Allele**	**Tm 1 (°C)**	**Tm 2 (°C)**	**Tm 3 (°C)**	**Average Tm (°C)**	**SD**
Blocker **4**	8	43,23	43,84	42,3	43,12	0,78
	9	48,44	48,65	48,12	48,40	0,27
	10	52,81	53,12	52,59	52,84	0,27
	11	55,85	56,04	55,63	55,84	0,21
	11+	57,41	57,6	57,29	57,43	0,16
Blocker **6**	11	49,08	49,29	49,28	49,22	0,12
	12	52,75	53,09	52,94	52,93	0,17
	13	56,16	56,32	56,48	56,32	0,16
	14	57,59	57,74	58,03	57,79	0,22

<u>Supplementary Table 10:</u> Melting temperatures of different alleles of the D16S59 locus using respectively the Bl4 and Bl6 blocker

**Amelogenin**	**Allele**	**Tm 1 (°C)**	**Tm 2 (°C)**	**Tm 3 (°C)**	**Average Tm (°C)**	**SD**
	X (♀)	50,58	51,73	51,08	51,13	0,58
	X (♂)	49,56	51,21	50,76	50,51	0,85
	Y	60,86	61,14	61,37	61,12	0,26

<u>Supplementary Table 11:</u> Melting temperatures of different alleles (XX- XY) of the amelogenin locus.
